# Hydrological Simulation for Predicting the Future Water Quality of Adyar River, Chennai, India

**DOI:** 10.3390/ijerph16234597

**Published:** 2019-11-20

**Authors:** Pankaj Kumar, Rajarshi Dasgupta, Manish Ramaiah, Ram Avtar, Brian Alan Johnson, Binaya Kumar Mishra

**Affiliations:** 1 Natural Resources and Ecosystem Services, Institute for Global Environmental Strategies, Hayama, Kanagawa 240-0115, Japan; dasgupta@iges.or.jp (R.D.); johnson@iges.or.jp (B.A.J.); 2 Graduate School of Environmental Science, Hokkaido University, Sapporo 060-0810, Japan; ramaiah.tuj@gmail.com (M.R.); ram@ees.hokudai.ac.jp (R.A.); 3 Faculty of Science and Technology, Pokhra University, Pokhra 56305, Nepal; bkmishra@pu.edu.np

**Keywords:** BOD, *Escherichia coli*, river pollution, wastewater management, water quality modelling, Chennai

## Abstract

Just a few decades ago, Adyar River in India’s city of Chennai was an important source of water for various uses. Due to local and global changes (e.g., population growth and climate change), its ecosystem and overall water quality, including its aesthetic value, has deteriorated, and the water has become unsuitable for commercial uses. Adverse impacts of excessive population and changing climate are expected to continue in the future. Thus, this study focused on predicting the future water quality of the Adyar river under “business as usual” (BAU) and “suitable with measures” scenarios. The water evaluation and planning (WEAP) simulation tool was used for this study. Water quality simulation along a 19 km stretch of the Adyar River, from downstream of the Chembarambakkam to Adyar (Bay of Bengal) was carried out. In this analysis, clear indication of further deterioration of Adyar water quality by 2030 under the BAU scenario was evidenced. This would be rendering the river unsuitable for many aquatic species. Due to both climate change (i.e., increased temperature and precipitation) and population growth, the WEAP model results indicated that by 2030, biochemical oxygen demand (BOD) and *Escherichia coli* concentrations will increase by 26.7% and 8.3%, respectively. On the other hand, under the scenario with measures being taken, which assumes that “all wastewater generated locally will be collected and treated in WWTP with a capacity of 886 million liter per day (MLD),” the river water quality is expected to significantly improve by 2030. Specifically, the model results showed largely reduced concentrations of BOD and *E. coli*, respectively, to the tune of 74.2% and 98.4% compared to the BAU scenario. However, even under the scenario with measures being taken, water quality remains a concern, especially in the downstream area, when compared with class B (fishable surface water quality desirable by the national government). These results indicate that the current management policies and near future water resources management plan (i.e., the scenario including mitigating measures) are not adequate to check pollution levels to within the desirable limits. Thus, there is a need for transdisciplinary research into how the water quality can be further improved (e.g., through ecosystem restoration or river rehabilitation).

## 1. Introduction

Water is intrinsically linked to the sustainable and inclusive development of human societies, with availability of clean and potable quality drinking water being a major global constraint [1,2] included the UN Sustainable Development Goals (SDGs). In fact, clean drinking water is not available to over 1.1 billion people, and it is feared that nearly 125 of 194 nations are projected to be water stressed by 2025 [3,4]. In fact, researchers have identified water insecurity as a major inhibitor of socio-economic growth in the developing world—particularly in Asia and Africa—with the increasing demand for water and the continued contamination of existing water sources further complicating the scenario [5,6]. Although the concept of water security varies within the existing scientific disclosures [7], most researchers now agree that the water crisis of the 21st century is much more related to improper water management rather than the actual scarcity of water [8]. Some pressing issues include the poor quality of potable water and lack of water governance in most of the developing countries, which broadened the debates on water security over the last two decades. Consequently, the SDGs simultaneously focused on water quantity and quality compared to the erstwhile Millennium Development Goals, which only provided quantity-related targets [9]. Nonetheless, rapid urbanization, population growth, and climate change also pose significant challenges to achieving future water security [5,6].

Of the several strategies discussed in the contemporary scientific disclosures, adaptive governance of water resources has been identified an important tool for achieving water security. However, management of urban aquatic ecosystems poses a significant challenge [10,11]; and the perception of urban water security is not the same as for the natural water resources of a nation [7]. A number of allied factors are particularly important in an urban context, including high population density, concentrated demand, distribution system, recycling of wastewater and taxation. To meet such complexity, tapping reliable, all-weather sources remains critical and the first step for urban water planners. This has often led to tapping potential sources far away from the cities and resulted in massive investments in water infrastructure. Yet, based on the 1960s’ per capita basis, there has been a drastic reduction, particularly in South Asia, in renewable water resources. By 2050, as many as three billion people are expected to locate to and reside in Asian cities [12]. In fact, by 2015 itself, the water stress was severe in Pakistan, Afghanistan and India, with Nepal and Bangladesh also reported to be water stressed [13]. Thus, meeting the SDGs of ensuring sustainable water supply in growing cities of Asia remains a critical challenge. Socio-economic factors, unfortunately, are not considered while planning for water resource management in cities, particularly in developing nations; it has been done largely in a piecemeal manner [14]. Urban water supply management in developing countries is often aimed solely at meeting the basic water demands through adequate supply. The vital step in this regard is integration of hydrological and socioeconomic factors for achieving future urban water security. Understanding urban water security through a systems perspective, including the natural (i.e., source), social, economic and infrastructural components, therefore, remains highly imperative.

Among a number of holistic approaches conceptualized for management of water resources since the 1980s, the integrated water resource management (IWRM) model has received the highest attention [7]. The IWRM model targets different components of water resource governance, including socio-economics, hydro-meteorology, industrial and agricultural practices, wastewater, etc., and thereby integrates them for science-led decision-making [15,16]. Several IWRM numerical models such as WEAP (water evaluation and planning), MIKE, RIBASIM (river basin simulation model) and WBalMo (water balance model) have been developed and widely used to address water security issues. Some of them are data intensive, and therefore remain unusable for data-deficit regions. The WEAP model is less data-intensive than most other models [17,18,19] and used is widely for modelling water quality in developing countries. Moreover, the software package comes free of cost, to the benefit of water resource planners.

A combination of high economic growth and haphazard and faster expansion of urban population has resulted in degradation of the water quality of many rivers, lakes, and coastal areas (e.g., due to increased pollution from household, industrial, and agricultural sources). This problem is particularly prominent in developing countries due to the previously mentioned inadequate water governance. Despite the importance of these water bodies, their health status and suitable steps for ensuring acceptable quality indices are poorly documented. In this study, we assessed the prevalent water quality and simulated its future trend by considering population growth, climate change and countermeasures in the Adyar River coursing through Chennai, one of the largest municipal economic centres of India. This will help to formulate steps for adequate management of this water resource.

## 2. Materials and Methods

### 2.1. Study Area

With an area of around 426 km^2^, Chennai—with a population of 7,088,000 [20], is the largest economic, educational and cultural center. It is located on the Coromandel Coast of the Bay of Bengal and is the capital city of Tamil Nadu situated in the northeastern part of the State [20]. The city is the fourth most populous urban agglomeration in India. The Chennai Metropolitan area (combination of Chennai district with Kancheepuram and Thiruvallur district) is one of the largest city economies of India. Chennai features a tropical wet and dry climate. The annual rainfall averages about 1400 mm. Late May and early June are the hottest periods, at 38–42 °C, and January the coolest, at 18–20 °C.

**Figure 1:**
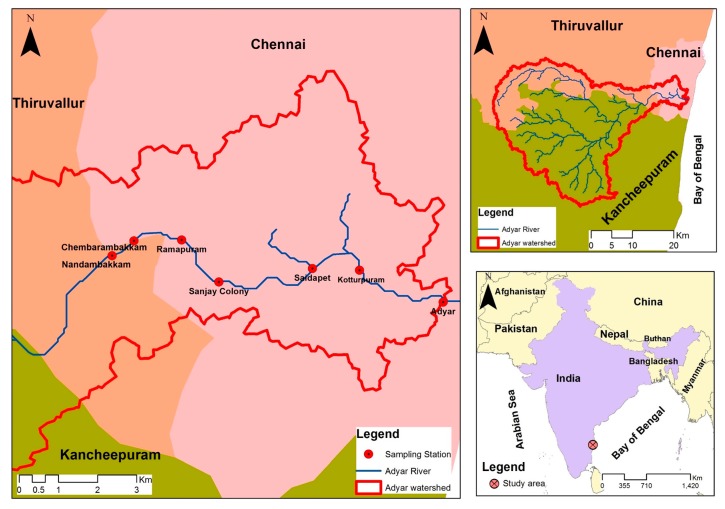
Study area map showing Adyar River passing across the south-central part of Chennai City.

Adyar River, which lies in the south of Chennai city, traverses it from west to east (Figure 1). The river is heavily polluted, limiting its usability as well as creating an unaesthetic ambient environment [21]. The river enters Chennai at Nandambakkam and traverses about 9 km through metropolitan area in a total of its 15 km stretch in the suburbs of Chennai. It ends as a wide lagoon—the Adyar estuary. In this estuarine region, there are large spans of sludge-filled backwaters leading to formation of many small islands.

### 2.2. Model Setup and Data Used

**Figure 2:**
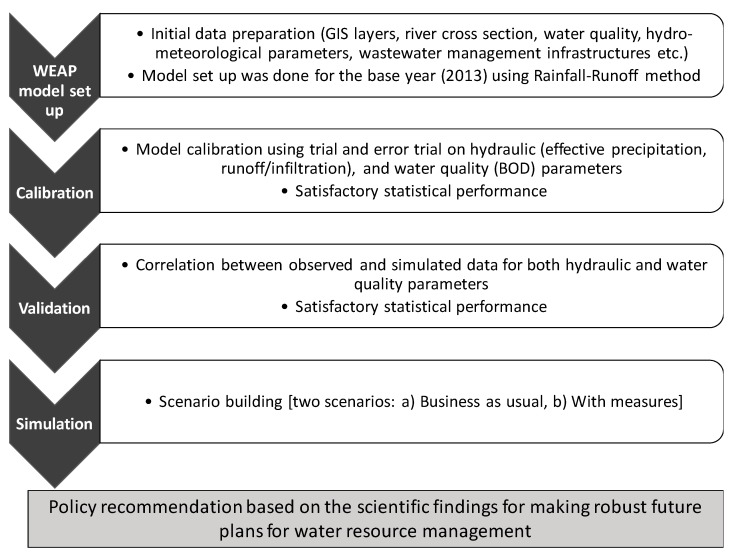
Flowchart showing work framework.

**Figure 3:**
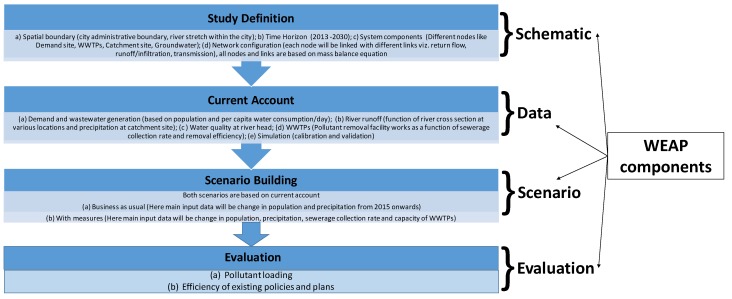
Flowchart showing model structure.

Based on the observed water quality data for six water samples for year 2013, a Piper diagram and a scatter plot was used to classify water samples into water types and to get deep insight. In order to assess alternative management policies in the Adyar River basin and for simulating future water quality variables in 2030, the WEAP model was set up. Here, year 2013, 2015 and 2030 were considered as the base year, current year and future target year respectively. The framework for the whole simulation is shown in Figure 2. The structure of the model is shown in Figure 3. A wide range of input data, namely, domestic waste water quality as a point source of pollution, past spatio-temporal river water quality, river length, river discharge and river flow-stage-width relationships [22], were used for water quality modeling. In addition, data on waste water treatment plants’ capacities and contaminant removal efficiencies, population [20], historical rainfall, evaporation, temperature [23] and drainage networks (percentage of households connected to main sewerage line) [24] were also used. Further, past land use/land cover maps (data from LANSAT series were used and downloaded from United States Geological Survey (http://earthexplorer.usgs.gov/)), and a city-level master plan which outlines countermeasures for improving future water environment were used [25].

The 1980 to 2016 data on daily rainfall collected by India Meteorology Department (IMD) at Chembarambakkam meteorological station were used. Further, the 2011–2016 IMD data of daily average stream flow measurements at Adyar, Kotturpuram, Saidapet, Sanjay Colony, Ramapuram and Chembarambakkam) were utilized for calibration and validation of the WEAP hydrology module simulation. The 2011–2015 data on biochemical oxygen demand (BOD) and total coliforms (taken as equivalent to *Escherichia coli* counts) collected at Kotturpuram, Saidapet, Sanjay Colony and Ramapuram stations were used for water quality modeling.

**Figure 4:**
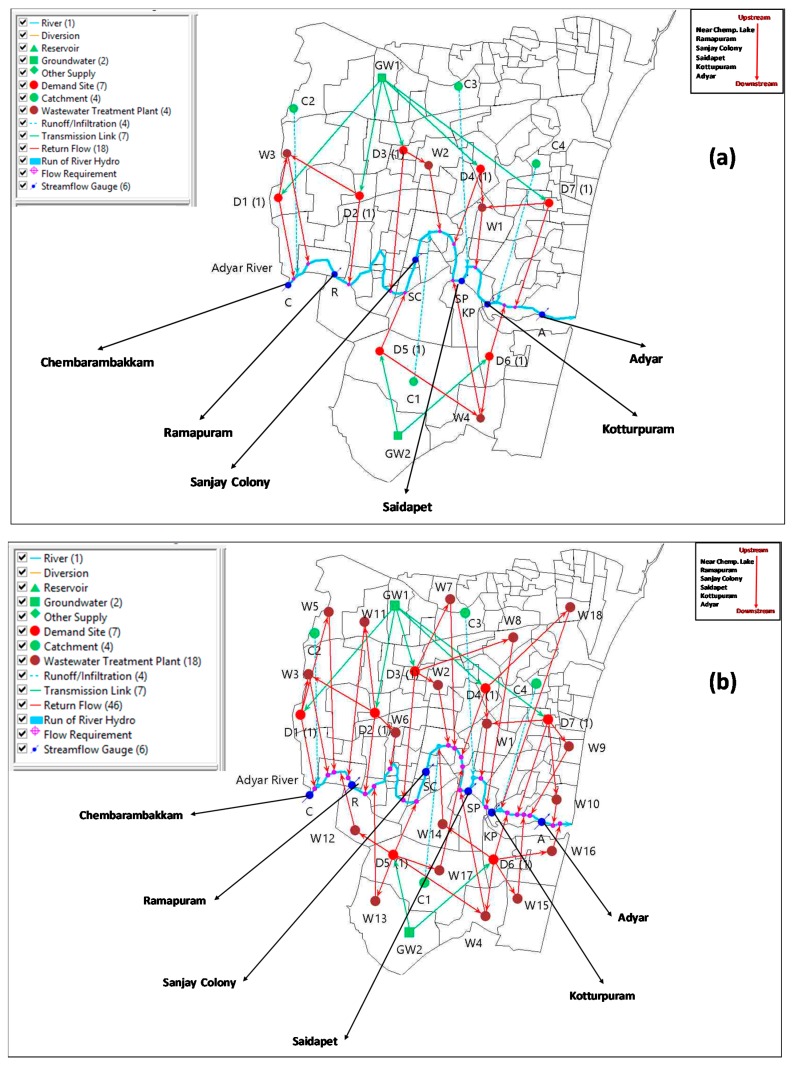
Schematic diagram highlighting the problem domain for modelling Adyar River water quality modeling using WEAP interfaces (**a**) for current year 2015 and (**b**) for future year 2030.

To develop the WEAP model for the Adyar River basin, for four catchment areas with inter-basin transmissions were considered. For the ease of modeling, the catchment areas were divided into six sub-catchments with considerations of physiography, the confluence points and climatic characteristics (Figure 4). The population distribution of sub-catchment areas was based on the ward zonation of Greater Chennai Corporation [26], as mentioned in Table 1.

Within the WEAP tool, the hydrology module enables quantification of catchment runoff which generates river discharge and pollutant transport dynamics with river flow. Water quality parameters that accumulate on catchment surfaces or drainage areas during non-rainy days reach water bodies through surface runoff. This hydrology module computes catchment surface pollutants generated as a product of time, runoff volume and proportion for different types of land use. During this study, the simulation of the land use information was broadly categorized into three categories; namely, agricultural, forest and built-up areas. These land use classes helped to determine infiltration runoff generated for that particular part of catchment area. The soil data parameters like hydraulic conductivity and porosity were identified using previous secondary data and the literature [27,28].

Knowledge of potential and adverse impacts of climate change is essential for developing adaptation as well as action measures to mitigate the ills of climate change [29]. Therefore, after downscaling and bias correction for future precipitation data, outputs of suitable global climate models (GCMs) for different representative concentration pathways (RCP) were used. Changes in monthly average precipitation were considered for evaluating the possible impacts of climate change on water quality. More importantly, to get climate variables at monthly scale, we aimed at providing a less computationally-demanding procedure for enabling the reduction of biases in the precipitation frequency and intensity [30]. Trend analyses on the basis of statistical downscaling were also considered. An analysis of 1980–2004 historical rainfall was done using the monthly precipitation data.

### 2.3. Model Setup

By taking into consideration of influent locations of major tributaries, the study area was divided into four catchments (Figure 4). To represent the problem domain in a holistic way, the other considerations being taken were seven demand sites and four wastewater treatment plant (WWTP). These demand sites basically represent domestic (population) centers with cumulative water consumed and wastewater pollution loads released to the river. Domestic wastewater was the only source of pollution considered in this work. Whereas, wastewater treatment plants with design specifications that include total capacity and pollutant removal rates are the only infrastructure to treat wastewater in the study area. Technology and contaminant removal efficiency of current WWTP are not reported anywhere. As per the Chennai City master plan, the WWTPs for the future are an up-flow anerobic sludge blanket reactor coupled with a sequencing batch reactor (UASB-SBR) [25]. Therefore, we have considered this same wastewater treatment technology and efficiency for both the current and future WWTPs. Also, as no precise data are available on total wastewater volume from domestic sources, the daily volume of domestic wastewater generation is taken as 180 liters equaling daily consumption per capita [22]. Once this model set up and calibration was completed, model validation was done using correlation analysis between the observed and simulated results of three-monthly average water quality tests for the current situation, i.e., 2015, at different locations and average river discharges during three rainy months for the years 2013 to 2015. Thereafter, numerical simulation worked out for business as usual (BAU) and mitigation measures scenarios. The WWTP capacity was assumed to be 180 MLD (total number = 4) for the current and BAU scenarios. This capacity was assumed to be 886 MLD18 numbers of WWTPs [25] for the scenario with mitigation measures.

## 3. Results

**Figure 5:**
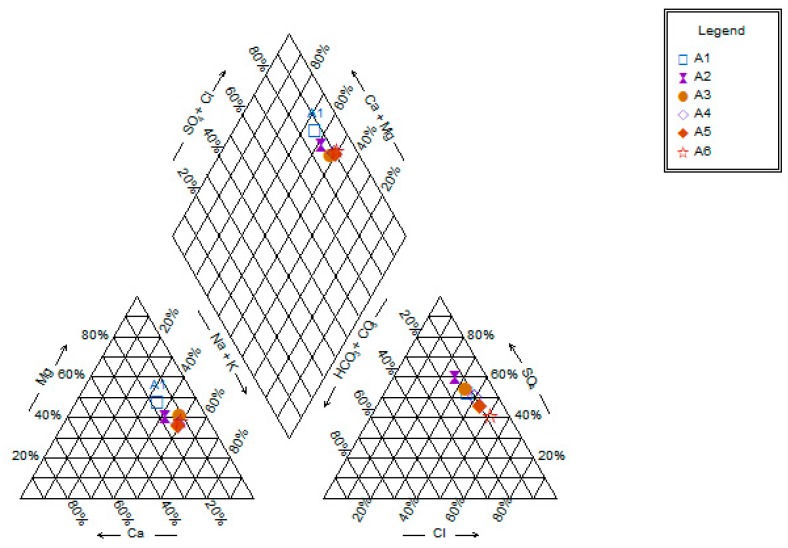
Piper diagram showing river water quality data for year 2013. A1—Chembarambakkam, A2—Ramapuram, A3—Sanjay Colony, A4—Saidapet, A5—Kotturpuram and A6—Adyar.

**Figure 6:**
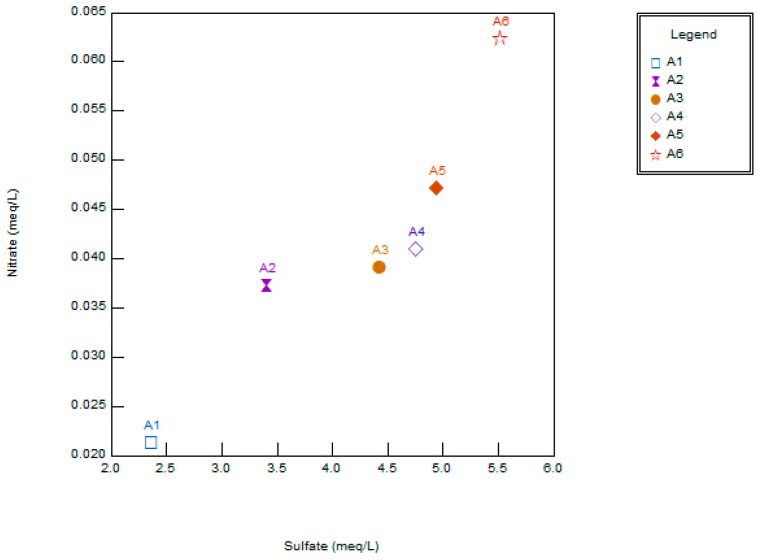
Scatter plot showing relationship between NO_3_^−^ and SO_4_^2−^.

Based on the piper diagram, water quality was analyzed for the year 2013 (Figure 5). All water samples fell under three water facies; i.e., NaSO_4_ (67%), MgSO_4_ (16%), and NaCl (17%). It depicts that water quality is being governed by both natural (rock-water interaction) and anthropogenic (domestic discharge, agricultural runoff, etc.). Further, a scatter plot between NO_3_^−^ and SO_4_^2−^ was made and the result is shown in Figure 6. It shows that concentrations for both NO_3_^−^ and SO_4_^2−^ are relatively higher except for Chembarambakkam at the upstream region. For the modeling, we selected only BOD and *E. coli*, because of its continuous data availability from 2013 to 2015.

In this study, there was a comprehensive assessment of possible climate change impacts on Adyar riverine ecosystem adopting MRI-CGCM3.2 and MIROC5 as GCMs with RCP4.5 and RCP8.5 emission scenarios. Both MRI-CGCM3.2 and MIROC5 were selected because of their wider use and higher resolution (120 Km) in South Asian region [31]. The RCPs are labeled according to the approximate global radiative-forcing level at 2100. RCP 4.5 was the normal emission scenario and 8.5 was the extreme emission scenario, which assumes that global annual GHG emissions (measured in CO_2_ concentration equivalents) continue to rise throughout the 21st century [32].

Future climate corresponds to the period of 2020–2044. The effect of population growth on water quality status was estimated by dividing the study area into seven demand sites. These sites represent population of settlements lying on both sides of the Adyar River. These settlements bear direct impact on the River by discharging domestic sewerage. Results for the population distribution and its future trend at four previously identified command areas were calculated by ratio method using UNDESA projected growth rate [12].

### 3.1. Precipitation Pattern

**Figure 7:**
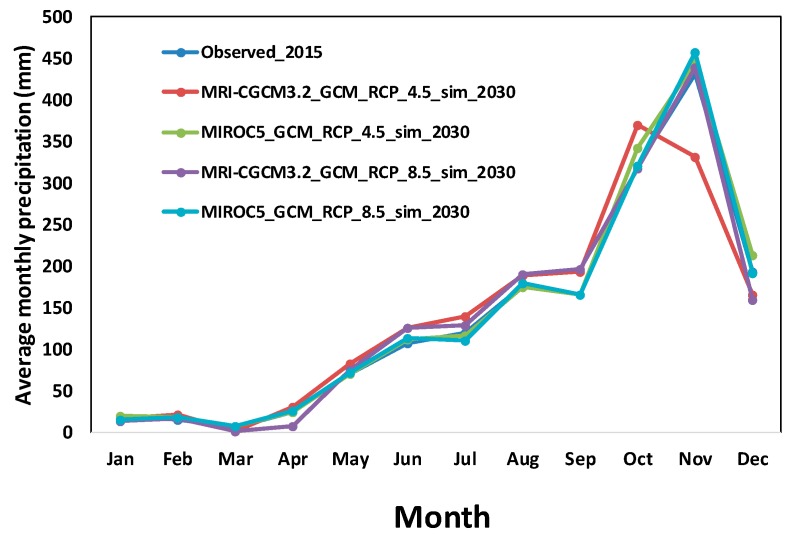
Patterns of projected monthly rainfall data, current and from models, at Chembarambakkam’s station.

The recorded annual precipitation for the year 2015 from IMD was 1652.6 mm, as depicted in Figure 7. Whereas, the projected annual precipitation values for MRICGCM3.2 GCM under RCP 4.5 and RCP 8.5 for the year 2030 were 1669.8 mm and 1676.3 mm, respectively. On the other hand, MIROC5 GCM using RCP 4.5 and RCP 8.5 projected precipitation values were 1715.5 mm and 1678.4 mm, respectively. Thus, projected annual precipitation simulated from GCM outputs is quite similar to the recorded IMD data. To further analyze the changes in precipitation, the graph is plotted between average monthly precipitation between the observed and simulated values (Figure 7).

### 3.2. Population Growth

The population figure of 5,196,533 was used for base year; i.e., 2013 in the study area (Census of India 2011). The annual growth rates 5% and 2.31% during the periods of 2013 to 2014 and 2015 to 2030 respectively (UNDESA 2015) were considered for projection of future population. Henceforth, populations considered for 2015 and target year (2030) were 5,446,829 and 7,672,113 respectively.

### 3.3. Water Quality 

#### 3.3.1. Model Performance Evaluation

**Figure 8:**
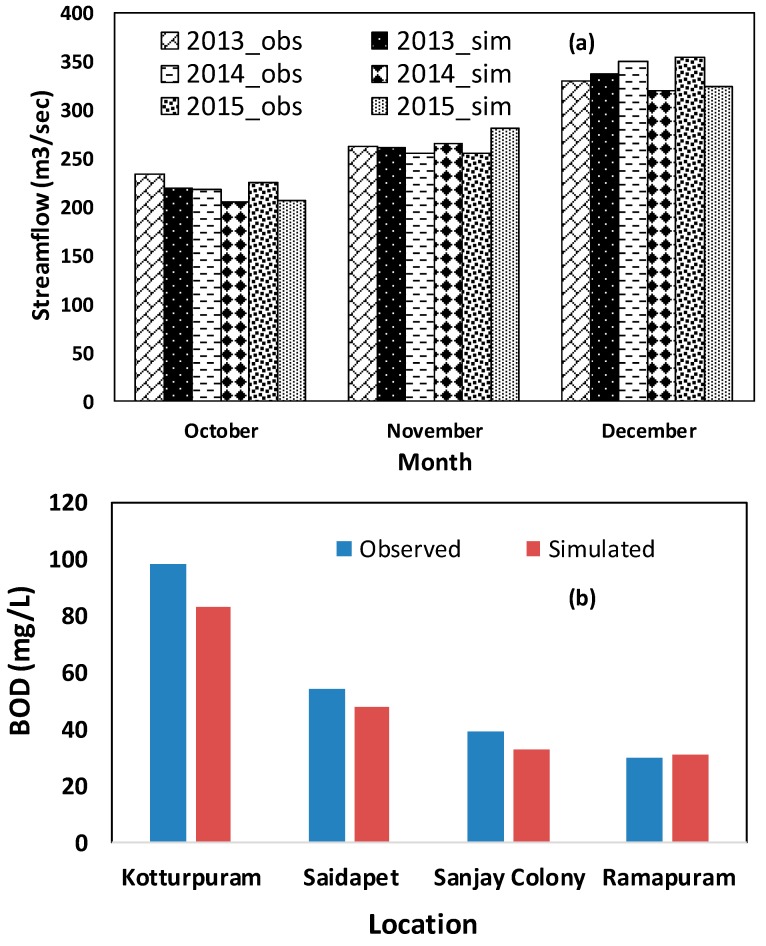
Comparison of simulated and observed (**a**) monthly river discharge at Sanjay Colony for years 2013–2015; and (**b**) the three months’ (October, November and December 2015) average biochemical oxygen demand (BOD) values at different locations.

As a first step, WEAP simulation performance was validated by comparing the result outputs from observed and simulated hydrological and water quality parameters. Many trial and error steps were run on two module parameters, which were effective precipitation and ratio of runoff to infiltration, for attaining proper simulation and reliable reproduction of the observed monthly stream flows for the period of year of 2013 to 2015. The final best fit parameters for both entities were 95% and 50/50 respectively. In Figure 8a, monthly simulated and observed stream flows at Sanjay Colony for years from 2103 to 2015 are presented. These largely match for most months with correlation coefficients (R^2^) ≅ 0.80, root-mean-square errors (RSMEs) ≅ 0.25 and an average error of 12%. October, November and December months were chosen for validation as no water is available in the river especially during these dry months. On the other hand, water quality output was also validated by relating three monthly (October, November and December) average simulated and observed BOD concentrations for the year 2015 at four different locations, as shown in Figure 8b. There is strong association between the observed and simulated values, as shown by average error 13%. Monthly average value was considered, as some of the values observed were not available at every time point. Year 2015 was chosen for the number of observed BOD values for the locations that were maximal. Strong relationship between the observed and simulated values for both monthly river discharges (Figure 8a) and BOD (Figure 8b), confirm suitability for model performance for both hydrological module and water quality module respectively. This study has following limitations: (a) the effect of dry and wet periods on the water quality parameters were not considered because of lack of data observed, (b) no seasonal fluctuation in per capita per day water consumption and domestic waste water generation was considered, which might cause a bias in simulated water quality when representing the real situation; (c) mixing of stormwater and waste water in the sewerage pipeline was not considered, which is highly likely during monsoon period, as the sewerage pipeline is a common carrier for both stormwater and domestic waste water in real situation.

#### 3.3.2. Scenario Analyses

Water quality simulation was done using two possible scenarios (Table 2) for the years 2015 and 2030 with 2013 as the reference year. Population increase; land use pattern changes; wastewater generation and treatment at wastewater treatment plants (WWTP); projected rainfall pattern; and all existing WWTPs, were also considered. The first scenario (the business as usual scenario considered), population-growth and climate change effects using the average value of two GCMs and two RCPs on water quality with the existing capacity of WWTPs of 180 MLD constants at year 2030. For a scenario with measures taken, all conditions were kept the same as first except for the enhanced WWTP capacity and collection rate (Table 2).

**Figure 9:**
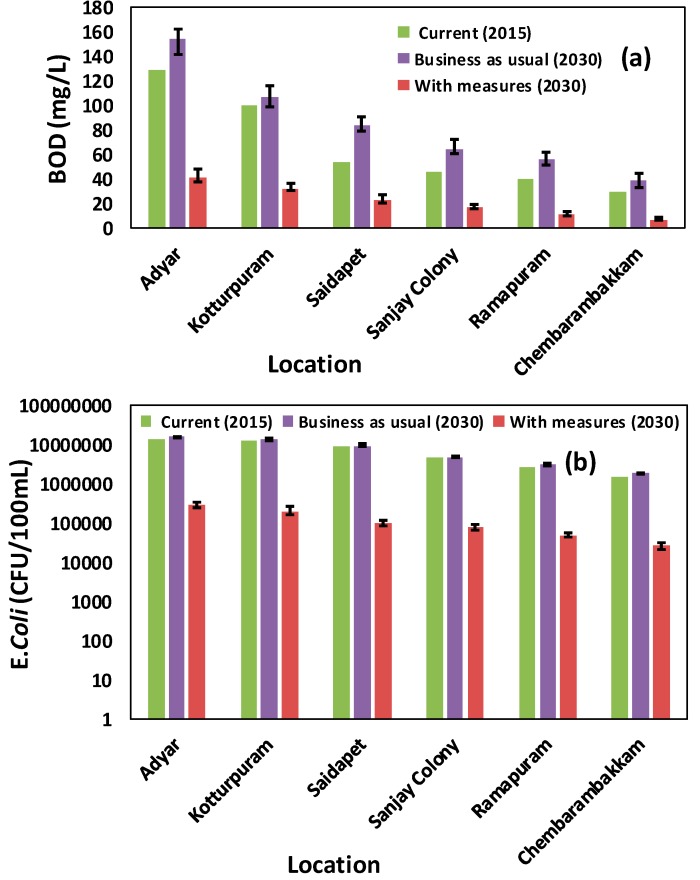
Simulated water quality parameters (**a**) BOD and (**b**) *Eschericia coli* for 2015, BAU (2030) and with measures (2030) scenarios.

In Figure 9, simulation results for BOD and *E*. *coli* counts using both scenarios are shown. Small bars on simulated water quality indicate the range due to change in GCM and RCP outputs. With the existing WWTP capacity of 180 MLD, in the present-day scenario, the water quality throughout the river is very poor. At the current capacity, the WWTPs are treating sewage from only 25% of total population in the study area. Thus, the water quality does not pass the local guidelines for class B (swimmable category (BOD < 3 mg/L and *E. coli* < 1000CFU/100 mL) [33]. The values of BOD observed for 2015 varied from 20 to 78 mg/L, suggesting extremely polluted waters which failed to fall within class B. The effects of both climate change and population change seem to prominently impact water quality status under the BAU scenario. It deteriorates further in 2030 with an average increase in BOD and *E. coli* loads by over 26.7% and 8.3% respectively. Using the individual effect of population growth, the value of rainfall as a representative of climate change by year 2030 kept constant or varied, indicating that population growth contributes highly to deterioration of water quality (Table 3) due to climate change. In the scenario with measures taken, where the whole wastewater generated locally will be collected and treated in a WWTP with a capacity of 886 MLD, that will reduce BOD and *E. coli* by 74.2% and 98.4% respectively, and improve water quality especially in the upper stretches of the River. However, based on the simulated value of two water quality parameters, as shown in Figure 9, water quality would still be a matter of concern in the downstream. Since installation of up-flow anerobic sludge blanket reactor coupled with sequencing batch reactor (UASB-SBR) type of WWTP was contaminant with the removal efficiency of 97% for BOD and 99.69% for fecal coliforms, they the best infrastructural features, suggested as per the existing master plan [34]. In addition, with their installation there will be a very high improvement in the quality of treated water, as the simulated result from this study suggests. These projections are useful for suggesting that a greater change in existing water management policies are needed to check the pollution levels. These simulated water quality results are also useful for pointing out the potential health risks of microbial contamination, algal blooms and the death of many aquatic organisms.

## 4. Conclusions and Recommendations

With a snapshot of the water quality status of Adyar River in Chennai City, India, from the present (2015), this study investigated future predictions (year 2030) using BAU and “with measures” scenarios using numerical simulation. With the actual water quality data, it was found that entire stretch of the Adyar River is already highly polluted as per the set standards of Tamil Nadu Pollution Control Board (TNPCB). Further, numerical simulation under the BAU scenario predicts that the water quality is bound to further deteriorate by 2030. Under the scenario including mitigation measures, the quality can improve significantly, except at downstream areas like Saidapet, Kotturpuram and Adyar, without adopting additional mitigation measures. Despite the considerable capacity of existing WWTPs, the wastewater not reaching these plants due to poor collection rates or poor connections between each household and the main sewerage line are currently the potential causes for very poor water quality. This is mainly due to unwillingness of local residents to pay the connection fee and subsequent water or sewerage treatment bills. Clogging of sewerage pipes, especially during rainy seasons as they carry both sewerage and stormwater, also adds to the problem. Despite many ministries (Water Resource Organization, Central Ground Water Board, Municipal Administration and Water Supply Department, etc.) being meant to manage water resources in the Chennai region, several of their efforts overlap, rather than being complementary. Improper coordination between different stakeholders in water management has caused the failure to implement the water infrastructure master plan in a timely manner. There are a lack of funds for regular operation and maintenance costs of existing structures and technical upgrades to the specifications of WWTPs.

To overcome many of these barriers, the first and foremost important thing is to do a diligent monitoring of water quality under the cradle to grave framework, i.e., from source of pollutant to the sink, which is necessary for building a reliable model to predict the best possible future water environment. This will include gathering accurate information about per capita daily intake of fresh water, including seasonal fluctuation; waste water generated with localities for the points of discharge; inventory for the WWTPs’ treatment capacity; and effluent quality. Lack of data is a common issue in developing nations, which hinders the precise assessment of future water quality in order to build a robust management plan. The other solutions are: create some political space where different stakeholders other than government agencies also have direct involvement in influencing the governing processes and government decisions; provide financial incentives to connect sewage of their households to the main sewerage line; and improve people’s awareness of the health and business (e.g., tourism) benefits of a better water environment.

As creating wastewater treatment facilities can be a financial burden for many in developing countries, the proposal of some business models for the operation and maintenance of sewerage lines and technical upgradation of existing WWTPs with a public-private-partnership model may create a win-win situation for every stakeholder. Here, we can consider willingness to pay of local people for getting a better water treatment and sanitation service as a matter of future research. In addition, a strong local government push to implement decentralized WWTPs along with the creation of centralized WWTPs must be considered. Locals and local government should be encouraged to maintain the existing septic tanks on a regular basis. On behalf of such practices, there should be some monetary incentives; e.g., through tax exemptions. For time bound completion of the exiting master plan, regular monitoring of the implementation-progress is highly mandatory. Finally, it can be said that such studies are necessary to advocate the policy planners at least at an advisory level to give an idea of the future status.

## Figures and Tables

**Table 1:** Summary of population distribution for catchments and sub-catchments considered for the simulation.

Catchment	Sub-Catchment	Ward Number	Growth@ 2.45% per Year	Growth@2.31% per Year	
			2013	2015	2030
C1	C1	1 to 33	985,073	1,032,520	1,454,353
C2	C2-1	34 to 63	1,055,685	1,106,533	1,558,603
	C2-2	64 to 93	906,639	950,309	1,338,554
C3	C3	94 to 126	717,314	751,864	1,059,035
C4	C4-1	127 to 142	670,854	703,166	990,443
	C4-2	143 to 157	860,968	902,438	1,271,125

**Table 2:** Summary of all the criteria considered for different scenarios in future water quality simulation.

Scenario	Components
Business as usual	Climate change + population growth +WWTP of 180 MLD
With measures	Climate change + population growth +WWTP of 886 MLD (100% collection rate)

**Table 3:** Summary of effect of individual parameters on simulated water quality.

Parameters	Average % Increase with Business as Usual Scenario (2015–2030)	% Contribution from Population Growth	% Contribution from Climate Change
BOD	26.7	87	13
*E. coli*	8.3	89	11
